# Combined Squalene and Tocotrienol-Rich Fraction Attenuates oxLDL-Induced Endothelial Dysfunction Through Modulation of NF-κB–Associated Inflammatory Signalling

**DOI:** 10.3390/life16101755

**Published:** 2026-10-20

**Authors:** Sahsikala Thangasparan, Yusof Kamisah, Nur Najmi Mohamad Anuar, Azizah Ugusman, Nurul ‘Izzah Ibrahim

**Affiliations:** 1 Department of Pharmacology, Faculty of Medicine, Universiti Kebangsaan Malaysia, Jalan Yaacob Latif, Bandar Tun Razak, Kuala Lumpur 56000, Malaysia; saseykala@gmail.com (S.T.); kamisah_y@hctm.ukm.edu.my (Y.K.); 2 Cardiovascular and Pulmonary Research Group, Universiti Kebangsaan Malaysia, Bangi 43600, Selangor, Malaysia; nurnajmi@ukm.edu.my (N.N.M.A.); dr.azizah@ppukm.ukm.edu.my (A.U.); 3 Programme of Biomedical Science, Center for Toxicology & Health Risk Studies (CORE), Faculty of Health Sciences, Universiti Kebangsaan Malaysia, Jalan Raja Muda Abdul Aziz, Kuala Lumpur 50300, Malaysia; 4 Department of Physiology, Faculty of Medicine, Universiti Kebangsaan Malaysia, Jalan Yaacob Latif, Bandar Tun Razak, Kuala Lumpur 56000, Malaysia

**Keywords:** squalene, tocotrienol-rich fraction, endothelial dysfunction, oxidised low-density lipoprotein, inflammation

## Abstract

Atherosclerosis is the principal underlying pathology of cardiovascular diseases (CVDs), with endothelial dysfunction representing one of the earliest events in its initiation and progression. Oxidised low-density lipoprotein (oxLDL) plays a pivotal role in this process by promoting inflammatory activation and endothelial dysfunction. Squalene and tocotrienol-rich fraction (SQTRF), both derived from palm oil, possess antioxidant and anti-inflammatory properties. However, their combined effects on endothelial dysfunction remain poorly understood. This study investigated the protective effects of SQTRF against oxLDL-induced endothelial dysfunction in human umbilical vein endothelial cells (HUVECs). HUVECs were exposed to oxLDL (50 μg/mL) and treated with SQTRF or TRF, with atorvastatin included as a positive control. Endothelial function was assessed by measuring cell viability, proliferation, migration, angiogenesis, and inflammatory gene expression using established in vitro assays and quantitative real-time PCR (qRT-PCR). OxLDL significantly enhanced endothelial migration and inflammatory gene expression without causing marked cytotoxicity. SQTRF preserved cell viability and significantly attenuated oxLDL-induced migration and angiogenesis. SQTRF demonstrated enhanced attenuation of endothelial proliferation and migration compared with TRF alone. These functional improvements were accompanied by downregulation of NF-κB, suggesting suppression of NF-κB-mediated inflammatory activation. Collectively, these findings demonstrate that SQTRF mitigates oxLDL-induced endothelial dysfunction through modulation of inflammatory and angiogenic responses. This study provides mechanistic evidence supporting the potential of SQTRF as a natural therapeutic candidate for attenuating early endothelial dysfunction associated with atherogenesis, warranting further validation in in vivo models and clinical studies.

## 1. Introduction

Cardiovascular diseases (CVDs) remain the leading cause of morbidity and mortality worldwide [1], with atherosclerosis-related disorders, including ischaemic heart disease and stroke, representing the primary underlying pathology [2]. Atherosclerosis is a chronic inflammatory disorder characterised by lipid accumulation, oxidative stress, and endothelial dysfunction, which collectively contribute to plaque development and vascular narrowing [3]. Endothelial injury represents a critical early event in atherogenesis, disrupting vascular homeostasis and promoting leukocyte recruitment and inflammatory activation [4].

Oxidised low-density lipoprotein (oxLDL) is a critical mediator involved in the initiation and progression of endothelial dysfunction during atherosclerosis [5]. Unlike native LDL, oxLDL possesses potent pro-inflammatory and pro-oxidative properties that promote endothelial activation and vascular injury [6]. The clinical relevance of oxLDL is supported by evidence that circulating oxLDL concentrations are higher in individuals with coronary artery disease than in healthy individuals. For example, Toshima et al. (2000) reported mean plasma oxLDL concentrations of 112.4 ± 3.3 U/dL in healthy controls compared with 201.3 ± 11.2 U/dL in patients with coronary heart disease [7]. Similarly, another study reported fasting plasma oxLDL concentrations of 218 ± 35 μg/L in healthy individuals and 575 ± 219 μg/L in patients with stable angina, with higher concentrations associated with greater coronary lesion severity [8]. Collectively, these findings indicate that circulating oxLDL is elevated in individuals with established coronary disease and may reflect systemic oxidative modification of LDL and atherosclerotic burden [9]. A major mechanism underlying oxLDL-mediated endothelial dysfunction involves interaction with scavenger receptors, particularly lectin-like oxidised LDL receptor-1 (LOX-1), which is highly expressed in activated endothelial cells [10]. Binding of oxLDL to LOX-1 induces oxidative stress generation and triggers downstream signalling pathways, including mitogen-activated protein kinase (MAPK) and nuclear factor kappa B (NF-κB) signalling, leading to endothelial inflammatory activation and functional impairment.

NF-κB is a key transcription factor regulating inflammatory responses in endothelial cells. Following activation, degradation of its inhibitory protein IκB permits translocation of NF-κB subunits, including p65/p50 heterodimers, to the nucleus, where they promote transcription of inflammatory genes. This activation enhances the expression of pro-inflammatory cytokines, including interleukin-1β (IL-1β), tumour necrosis factor-α (TNF-α), and interleukin-6 (IL-6), as well as endothelial adhesion molecules such as vascular cell adhesion molecule-1 (VCAM-1), intercellular adhesion molecule-1 (ICAM-1), and E-selectin [11]. These molecular events link oxLDL exposure to endothelial inflammation and dysfunction, highlighting its fundamental role in the pathophysiology of atherosclerosis. In addition to inflammatory activation, oxLDL also disrupts essential endothelial functions involved in vascular maintenance and repair, including cell viability, proliferation, migration, and angiogenic capacity. Previous studies have demonstrated that oxLDL exposure reduced cell viability [12], increased cell proliferation [13], and increased cell migration in endothelial cells [14].

Consequently, increasing attention has been directed toward natural bioactive compounds with anti-inflammatory and vascular protective properties as potential complementary approaches for preventing endothelial dysfunction and atherosclerosis. Squalene (SQ), a naturally occurring triterpenoid found in shark liver oil, olives, wheat germ, rice bran, amaranth, and palm [15], exhibits anti-inflammatory and lipid-modulating activities that may contribute to vascular protection [16]. Although shark liver oil has traditionally been considered a major source of squalene, concerns regarding sustainability, animal conservation, and potential environmental contaminants have encouraged the exploration of alternative plant-derived sources. SQ is naturally present in plant oils such as olive oil, palm oil, amaranth oil, and vegetable oil [17]. Among these sources, palm fatty acid distillate (PFAD), a by-product of palm oil refining, has emerged as a valuable source of squalene, containing approximately 415 ± 5 ppm [18], and has been recognised as a promising valuable source of squalene [19]. In addition to SQ, PFAD contains significant amounts of extractable lipid-soluble compounds such as tocopherols, tocotrienols, and phytosterols. Although SQ is susceptible to oxidation due to its highly unsaturated structure, it occurs naturally within lipid matrices together with other bioactive lipid-soluble compounds. In this study, SQ was evaluated in combination with tocotrienol-rich fraction (TRF), rather than as a purified compound or its hydrogenated derivative, squalane, to investigate the potential complementary biological effects of SQ and tocotrienols. Tocotrienols are members of the vitamin E family that can be naturally extracted from palm oil, rice bran oil, coconut oil, barley germ, and annatto [20,21]. They have demonstrated antioxidant, anti-inflammatory, and cardioprotective properties in experimental studies [22,23].

Importantly, circulating concentrations of SQ and tocotrienols in humans provide useful context to interpret the potential biological relevance of the concentrations investigated in vitro. Fasting serum SQ concentrations have been reported at approximately 163 ± 11 μg/dL in healthy adults, with SQ distributed predominantly among circulating lipoproteins. Following oral administration of 500 mg SQ, circulating SQ increased significantly in triglyceride-rich lipoprotein fractions, including chylomicrons and very-low-density lipoproteins (VLDLs), demonstrating that dietary SQ can influence circulating exposure [24]. In contrast, tocotrienols are present at considerably lower circulating concentrations in fasting conditions. In a pharmacokinetic study in healthy volunteers reported that a single oral dose of 526 mg palm-derived TRF resulted in peak plasma concentrations of 4.74 ± 1.69 μM α-tocotrienol, 2.73 ± 1.27 μM γ-tocotrienol, and 0.53 ± 0.25 μM δ-tocotrienol at approximately four to five hours after supplementation, while tocotrienols were undetectable in fasting plasma [25]. Following longer-term supplementation, daily consumption of 200 mg tocotrienol preparation for 8 weeks significantly increased plasma tocotrienol concentrations. In the tocotrienol-supplemented group, plasma α-tocotrienol increased from 9.9 ± 2.5 ng/mL at baseline to 112.8 ± 38.0 ng/mL, γ-tocotrienol from 19.2 ± 3.1 to 161.7 ± 49.9 ng/mL, and δ-tocotrienol from 2.5 ± 0.9 to 17.9 ± 4.7 ng/mL after 8 weeks of supplementation. These concentrations returned to baseline following a six-week washout period [26]. Collectively, these findings demonstrate that circulating exposure to SQ and tocotrienols varies according to dietary intake, supplementation dose, formulation, and timing of blood sampling. Although direct comparison between circulating concentrations and in vitro experimental concentrations is limited by differences in pharmacokinetics, lipoprotein distribution, protein binding, and cellular uptake, these human data provide a useful reference framework for interpreting the potential translational relevance of the present findings.

Although the anti-atherogenic effects of squalene from shark liver oil and plant sources, as well as tocotrienols, have been extensively reported, evidence regarding the protective effects of their combined formulation on oxLDL-induced endothelial dysfunction remains scarce. While the antioxidant properties of tocotrienols have been well established, the anti-inflammatory effects of the combined squalene and tocotrienol-rich fraction (SQTRF) preparation in relation to NF-κB-associated endothelial activation remain less well characterised. As NF-κB is a key regulator of endothelial inflammatory responses and promotes the expression of adhesion molecules such as intercellular adhesion molecule-1 (ICAM-1) and vascular cell adhesion molecule-1 (VCAM-1), modulation of these markers may provide insight into the inflammatory component of oxLDL-induced endothelial dysfunction. Whether combined SQTRF attenuates endothelial dysfunction through modulation of NF-κB signalling, adhesion molecule(ICAM-1 and VCAM-1), and endothelial functional responses has not been fully elucidated. Therefore, this study investigated the protective effects of palm-derived SQTRF against oxLDL-induced endothelial dysfunction in human umbilical vein endothelial cells (HUVECs).

## 2. Materials and Methods

### 2.1. Isolation of Human Umbilical Vein Endothelial Cells (HUVECs)

Primary human umbilical vein endothelial cells (HUVECs) were isolated via collagenase digestion from umbilical cord veins obtained from healthy full-term deliveries at Hospital Canselor Tuanku Muhriz, Cheras, Kuala Lumpur, Malaysia according to a procedure as previously described by Suhaila and Safuan (2017) [27]. All procedures involving human samples were conducted in accordance with the ethical guidelines approved by the Universiti Kebangsaan Malaysia Ethical Committee (UKM PPI/111/8/JEP-2020-006). Written informed consent was obtained from all donors prior to sample collection.

### 2.2. Cell Treatment

HUVECs were routinely cultured in endothelial growth medium at 37 °C in a humidified incubator with 5% CO_2_. Endothelial identity was confirmed based on characteristic cobblestone morphology and positive CD31 expression using immunocytochemistry. The culture medium was replaced every 2 days, and cells at passages three to five were used for all experiments [28]. OxLDL ( Invitrogen by Thermo Fisher Scientific, Eugene, OR, USA) was prepared using serum-free media (SFM) and used to establish an in vitro model of oxLDL-induced endothelial dysfunction. HUVECs were exposed to different concentrations (20, 50, and 100 μg/mL) to determine the optimal concentration for subsequent experiments. SQTRF and TRF were obtained from Phytogaia Sdn. Bhd., Kuala Lumpur, Malaysia and were mixed with 0.05% ethanol and SFM at a 1:1 ratio. Following optimisation of the oxLDL concentration, HUVECs were exposed to oxLDL prior to treatment with SQTRF for 24 h. SQTRF was evaluated at concentrations ranging from 8.2 to 131.2 μg/mL based on a previous study [29]. TRF was used at 4.11 μg/mL based on previous experimental findings [30]. Atorvastatin (10 μM) was included as a positive control, with this concentration selected based on previously a published in vitro study investigating atorvastatin in endothelial cells [31]. No independent dose-optimisation experiments were performed for either TRF or atorvastatin in the present study.

### 2.3. Optimisation of oxLDL and SQTRF Concentrations

Preliminary concentration optimisation experiments were performed to determine the appropriate concentrations of oxLDL and SQTRF for subsequent experiments in HUVECs. Cells were exposed to oxLDL (20, 50, and 100 µg/mL) for 24 h, and endothelial angiogenic function was assessed using a tube formation assay. The optimal oxLDL concentration was selected based on its ability to induce endothelial dysfunction while maintaining cellular integrity.

For SQTRF concentration optimisation, HUVECs were treated with increasing concentrations of SQTRF (8.2 to 131.2 µg/mL) for 24 hours, and cell viability was assessed using the MTT assay. Concentrations that maintained ≥90% cell viability were considered non-cytotoxic and suitable for subsequent experiments.

### 2.4. Assessment of Cell Viability

Cell viability was assessed using the MTT assay. Briefly, HUVECs were seeded in 96-well plates (5 × 10^3^ cells per well) and were incubated overnight to allow attachment. Following the cell seeding, the cells were treated with SQTRF, TRF and/or oxLDL and incubated for 24 h. Then, MTT solution (5 mg/mL) was added to each well, and the plates were incubated for 4 h at 37 °C. The media was carefully removed, and the resulting formazan crystals in each well were solubilised in dimethyl sulfoxide. The absorbance was measured at 570 nm using a microplate reader. Cell viability was expressed as a percentage relative to untreated control cells.

### 2.5. Assessment of Tube Formation Assay (Angiogenesis)

The angiogenic capacity of HUVECs was evaluated using a Matrigel tube formation assay. Briefly, growth factor-reduced Matrigel (Corning, NY, USA) was thawed on ice, and 10 μL was carefully dispensed into each well of a 15-well μ-Slide (Ibidi, Germany) without introducing air bubbles. The Matrigel was allowed to polymerise for 1 h at 37 °C in a humidified incubator with 5% CO_2_.

Following treatment according to the experimental groups, HUVECs were harvested and seeded onto the Matrigel-coated wells at a final density of 2 × 10^5^ cells/mL in endothelial growth medium. Cells were incubated at 37 °C in a humidified atmosphere containing 5% CO_2_, and tube formation was monitored every 2 h for up to 6 h using an inverted phase-contrast microscope. Representative images acquired at the 6 h time point were analysed using the Angiogenesis Analyzer plugin in ImageJ version 1.54. Angiogenic activity was quantified by determining the mean number of branch points. Each experiment was performed in triplicate.

### 2.6. Assessment of Cell Proliferation (BrdU Assay)

Cell proliferation was assessed using a bromodeoxyuridine (BrdU) Cell Proliferation Assay Kit (Merck, Darmstadt, Germany) according to the manufacturer’s instructions. Briefly, HUVECs were seeded into 96-well plates at a density of 1 × 10^5^ cells/mL (100 μL/well) and cultured for 24 h until approximately 80% confluence was reached. Blank wells containing culture medium only and background control wells without BrdU labelling were included to validate the assay.

Following treatment according to the experimental groups, cells were incubated for 4 h at 37 °C in a humidified atmosphere containing 5% CO_2_. BrdU labelling solution was then added to each well, and the cells were incubated for a further 20 h to allow BrdU incorporation into newly synthesised DNA. Cells were subsequently fixed and the DNA denatured using the fixation/denaturing solution provided in the kit. Incorporated BrdU was detected using a mouse anti-BrdU monoclonal antibody followed by horseradish peroxidase (HRP)-conjugated goat anti-mouse IgG. After substrate development, the reaction was terminated with stop solution, and absorbance was measured at 450–540 nm using a microplate reader. Cell proliferation was expressed as the relative absorbance normalised to the untreated control group. All experiments were performed in triplicate.

### 2.7. Assessment of Cell Migration Wound Healing Assay (Migration)

The migratory ability of HUVECs was evaluated by an in vitro wound healing scratch assay. Briefly, 100 µL HUVECs were seeded at a density of 1 × 10^6^ cells/mL in 24-well plates and cultured in endothelial cell growth medium (ECM) until an approximately 80% confluent monolayer was achieved. Prior to scratch induction, the culture medium was removed, and the cells were gently washed with phosphate-buffered saline (PBS).

A linear scratch wound was created across the cell monolayer using a sterile 200 μL pipette tip. Following scratch formation, the detached cells were removed by washing with PBS, and the cells were subsequently treated with oxLDL and/or SQTRF. The wound closure process was monitored for 24 h using an inverted microscope under time-lapse imaging conditions.

Images were captured at baseline (0 h) and after 24 h of treatment. The changes in wound area were quantified using ImageJ software, and the wound closure rate was calculated using the following formula:

$$\text{Migration rate/Wound closure rate }=\frac{Wound\;Length\;at\;0\;h-Wound\;Length\;at\;respective\;h}{Duration\;at\;the\;respective\;h}$$

### 2.8. Assessment of Gene Expression Analysis (qRT-PCR)

The mRNA expression levels of endothelial adhesion molecules including VCAM-1, ICAM-1, and the inflammatory transcription factor NF-κB p65 were evaluated by quantitative real-time polymerase chain reaction (qRT-PCR).

#### 2.8.1. RNA Extraction and cDNA Synthesis

Following treatment with oxLDL and/or SQTRF for 24 h, total RNA was extracted from HUVECs using a 1st Base PrimeWay Total RNA Extraction Kit (1st BASE, Singapore) according to the manufacturer’s instructions. Briefly, RNA was isolated using a silica membrane-based spin column method with DNase treatment to remove genomic DNA contamination. The concentration and purity of the extracted RNA were determined using a NanoDrop ND-2000 C spectrophotometer (Thermo Fisher Scientific, Waltham, MA, USA), and purified RNA samples were stored at −80 °C until further analysis.

Complementary DNA (cDNA) was synthesised from extracted RNA using ReverTra Ace™ qPCR RT Master Mix (TOYOBO, Osaka, Japan) with gDNA Remover according to the manufacturer’s protocol. The reverse transcription reaction was performed following genomic DNA removal, and the generated cDNA was stored at −20 °C prior to qRT-PCR analysis.

#### 2.8.2. Quantitative Real-Time PCR Analysis

Quantitative PCR was performed using THUNDERBIRD™ SYBR™ qPCR Mix (TOYOBO, Osaka, Japan) in a 384-well PCR plate. Each reaction mixture contained SYBR Green master mix, forward and reverse primers, cDNA template, and RNase-free water in a final reaction volume of 10 μL. No-template controls (NTCs) and no-reverse transcriptase controls (NRTs) were included to ensure the specificity of amplification and absence of genomic DNA contamination.

The amplification protocol consisted of an initial denaturation step at 95 °C for 3 min, followed by 40 amplification cycles of denaturation at 95 °C for 15–30 s and primer annealing at 60 °C for 30 s. A melting curve analysis was performed to confirm amplification specificity. Relative mRNA expression levels were determined from the threshold cycle (Ct) values after normalisation to glyceraldehyde-3-phosphate dehydrogenase (GAPDH), which served as the endogenous reference gene. Primer sequences for VCAM-1, ICAM-1, NF-κB p65, and GAPDH are listed in Table 1.

### 2.9. Statistical Analysis

All experiments were performed in three independent biological replicates (n = 3), with three technical replicates for each biological replicate. Data are presented as mean ± standard deviation (SD). Normality of data distribution was assessed using the Shapiro–Wilk test. For normally distributed data, one-way or two-way analysis of variance (ANOVA) was performed, followed by Tukey’s post hoc test for multiple comparisons. A *p*-value < 0.05 was considered statistically significant. Statistical analyses were performed using IBM SPSS Statistics version 27.0.

## 3. Results

### 3.1. Optimisation of oxLDL and SQTRF Concentrations for Endothelial Experiments

The optimal concentration of oxLDL for inducing endothelial dysfunction was determined based on its effects on endothelial tube formation. Exposure of HUVECs to oxLDL resulted in significant alterations in endothelial tube formation compared with the control group (Figure 1a). Among the tested concentrations, 50 µg/mL oxLDL produced the greatest effect on angiogenic activity, as demonstrated by the highest mean number of branch points (*p* < 0.05). Although 100 µg/mL oxLDL also disrupted tube formation, this concentration was associated with reduced network stability and compromised cellular integrity, suggesting a potential cytotoxic effect. Therefore, 50 µg/mL oxLDL was selected for subsequent experiments, as it effectively induced endothelial dysfunction while preserving sufficient cellular integrity for evaluation of the protective effects of SQTRF.

Following establishment of the oxLDL-induced endothelial dysfunction model, the cytotoxicity profile of SQTRF was evaluated. HUVECs treated with increasing concentrations of SQTRF (8.2, 16.4, 32.8, 49.2, 65.6, and 131.2 µg/mL) for 24 h showed a concentration-dependent cytotoxic effect. Lower concentrations of SQTRF (8.2, 16.4, and 32.8 µg/mL) maintained cell viability between 99% and 100%. In contrast, exposure to 131.2 µg/mL SQTRF significantly reduced HUVEC viability to 52.46% (*p* < 0.05), indicating a cytotoxic threshold at higher concentrations (Figure 1b). Therefore, non-cytotoxic concentrations of SQTRF (8.2, 16.4, and 32.8 µg/mL) were selected for subsequent experiments to investigate its protective effects against oxLDL-induced endothelial dysfunction.

### 3.2. SQTRF Protects Against oxLDL-Induced Endothelial Dysfunction

SQTRF exerted protective effects against oxLDL-induced endothelial dysfunction across multiple functional parameters. In the cell viability assay, oxLDL exposure did not significantly affect HUVEC viability compared with the control group. Treatment with SQTRF at a high concentration preserved cell viability and significantly increased cell viability compared with the control group (Figure 2a). OxLDL significantly enhanced migratory activity compared to control group. Although oxLDL also increased angiogenic activity and cell proliferation, these changes did not reach statistical significance. Treatment with SQTRF significantly attenuated these effects by reducing tube formation, cell proliferation, and migration compared with the oxLDL group (Figure 2b–d). These findings indicate that SQTRF mitigates functional alterations induced by oxLDL in endothelial cells. Representative images of the capillary-like tube networks are shown in Figure 3, illustrating the effects of oxLDL and SQTRF treatment on endothelial tube formation at the 2 h, 4 h, and 6 h time points. Representative images of the scratch wounds are shown in Figure 4, illustrating the effects of oxLDL, SQTRF, and atorvastatin on HUVEC migration at 0, 6, 12, and 24 h following scratch creation.

### 3.3. SQTRF Modulates Adhesion Molecules and Attenuates NF-κB Signalling Expression in oxLDL-Induced HUVECs

Treatment with SQTRF alone significantly increased VCAM-1 mRNA expression compared to the control. In oxLDL-exposed cells, co-treatment with SQTRF, atorvastatin, or TRF reduced VCAM-1 expression relative to the oxLDL group; however, these reductions did not reach statistical significance under any treatment condition (Figure 5a).

OxLDL alone and SQTRF alone significantly increased ICAM-1 mRNA expression compared with the control group (*p* < 0.05). Similarly, co-treatment with oxLDL and SQTRF also resulted in elevated ICAM-1 expression relative to the control. In contrast, co-treatment with atorvastatin or TRF significantly reduced ICAM-1 expression compared with the oxLDL group (*p* < 0.05) (Figure 5b).

Exposure to OxLDL alone and SQTRF alone significantly upregulated NF-κB mRNA expression compared with the control group (*p* < 0.05). Co-treatment with SQTRF, atorvastatin, or TRF significantly reduced NF-κB expression in oxLDL-stimulated cells compared with the oxLDL group (*p* < 0.05). The magnitude of NF-κB suppression observed with SQTRF was comparable to that of atorvastatin and TRF (Figure 5c).

## 4. Discussion

Activation of inflammatory pathways and endothelium dysfunction are pivotal events in the initiation and progression of atherosclerosis. Elevated circulating levels of low-density lipoprotein (LDL) are strongly associated with increased atherosclerotic burden and disease severity. LDL particles are susceptible to oxidative modification within the vascular wall, leading to the formation of oxidised LDL (oxLDL) [5]. OxLDL plays a critical role in the pathogenesis of atherosclerosis by promoting endothelial activation, inflammatory signalling, and vascular dysfunction [32]. OxLDL is widely used to induce endothelial dysfunction and simulate early atherogenic events in vitro [33,34]. In the present study, 50 μg/mL oxLDL was selected as the working concentration, as it produced the most pronounced disruption of angiogenic activity without compromising cell integrity. Interestingly, angiogenic activity was reduced at 100 μg/mL compared with 50 μg/mL. This attenuation at the higher concentration may reflect the onset of cytotoxic or pro-apoptotic effects, which could compromise endothelial functional responses. Consistent with our findings, Zhu et al. (2022) reported that 50 μg/mL oxLDL was optimal for establishing an in vitro atherosclerosis model compared with 10, 20, 100, and 200 μg/mL [33]. Although higher concentrations such as 100 μg/mL have also been employed in a previous study [34], the biological response to oxLDL appears to be concentration-dependent and may vary according to experimental endpoints and exposure duration.

Prior to evaluating its protective effects, the cytotoxic profile of SQTRF at low to high concentrations (8.2–131.2 μg/mL) was assessed to ensure biological safety within the experimental range. SQTRF at low to medium concentrations (8.2–32.8 μg/mL) maintained endothelial cell viability comparable to untreated controls, indicating that SQTRF is not essentially cytotoxic under the conditions tested. This finding confirms that the functional improvements observed in angiogenesis, migration, and proliferation assays are unlikely to be secondary to cell death. The selection of these concentrations for subsequent experiments was therefore biologically justified and allowed the anti-atherogenic effects of SQTRF to be evaluated independently of cytotoxic interference.

In the present study, oxLDL exposure did not significantly compromise HUVEC viability, indicating that the model represents early-stage endothelial dysfunction characterised by functional alterations rather than cytotoxicity or loss of cell integrity. This observation is consistent with previous studies demonstrating that low-to-moderate concentrations of oxLDL promote endothelial activation, migration, and angiogenic signalling without immediately inducing apoptosis [35]. In addition, oxLDL has been demonstrated to exert a biphasic effect on angiogenesis, enhancing neovascular responses at low concentrations but becoming inhibitory at high concentrations where cytotoxicity predominates [36].

Apart from that, when evaluating potential therapeutic agents in endothelial models, it is important to ensure that the results are not confounded by cytotoxicity. In the present study, SQTRF at low to medium concentrations preserved endothelial viability and, at higher concentrations, maintained cell survival at a level comparable to or greater than the control group. This indicates that SQTRF is not cytotoxic under the conditions tested. The absence of cytotoxicity suggests that the inhibitory effects of SQTRF on oxLDL-induced tube formation, migration, and proliferation are unlikely to be secondary to cell death, but rather reflect targeted modulation of endothelial signalling pathways.

The tube formation assay is widely recognised as a standard in vitro method for evaluating angiogenic potential. The formation of capillary-like networks by endothelial cells on a basement membrane matrix mimics key steps of the angiogenic process observed in vivo [37]. Angiogenesis plays a critical role in both physiological and pathological conditions, including tissue repair, tumorigenesis, and atherosclerosis, with its regulatory mechanisms and biological consequences varying depending on the disease context [38]. In atherosclerosis, angiogenesis occurs predominantly within atherosclerotic plaques and is considered a pathological event. Although the formation of new blood vessel initially serves to alleviate hypoxia within the expanding plaque, these immature and fragile microvessels contribute to intraplaque haemorrhage, inflammation, and increased risk of plaque instability and rupture [39]. In the present study, SQTRF treatment significantly reduced tube formation in oxLDL-induced HUVECs compared to the oxLDL-treated group. Notably, this inhibitory effect occurred in the absence of cytotoxicity, suggesting that SQTRF does not suppress endothelial viability, but rather modulates oxLDL-driven pro-angiogenic activation. Thus, the observed reduction in tube formation may reflect normalisation of pathological angiogenic signalling induced by oxLDL, rather than a general inhibition of physiological angiogenesis. These findings support the potential role of SQTRF in mitigating pathological angiogenic responses associated with early atherogenesis.

During atherosclerosis development, endothelial cell proliferation and migration are central to angiogenesis. The formation of new capillary networks from pre-existing vessels requires coordinated endothelial sprouting, migration, and proliferation, through both sprouting and intussusceptive mechanisms. Among these, sprouting angiogenesis, which can be characterised by endothelial budding and branching from existing vessels, predominates in the formation of microvascular plexuses and contributes significantly to intraplaque neovascularisation in atherosclerotic lesions [40]. In the present study, SQTRF treatment significantly reduced endothelial cell proliferation in oxLDL-induced HUVECs compared with the oxLDL-treated TRF group. This finding suggests that the combination of squalene and tocotrienol-rich fraction may exert enhanced modulatory effects on the oxLDL-induced HUVECs. The greater reduction observed with SQTRF compared to TRF alone indicates a potential additive or synergistic interaction between squalene and tocotrienols in attenuating pathological endothelial proliferation. To support this present study, an older experimental study by Khor and Chieng (1997) using hamster model showed that tocotrienol supplementation enhanced the cholesterol-lowering effects of dietary squalene compared with squalene alone, suggesting beneficial combined actions in vivo [41].

Endothelial cell migration is a key pathological process in atherosclerosis, contributing to plaque progression and instability. In response to disturbed shear stress and inflammation, endothelial cells undergo endothelial-to-mesenchymal transition (EndMT), increasing vascular permeability and leukocyte infiltration. Migratory endothelial cells also promote intraplaque neovascularisation, predisposing plaques to haemorrhage and rupture [42]. In the present study, oxLDL treatment significantly increased the migratory capacity of HUVECs compared with the control group. This observation is consistent with the findings of Ma et al. (2024) [43], who reported that oxLDL significantly increased the number of migrated endothelial cells per field. The authors further postulated that this enhanced migratory behaviour was mediated by induction of endothelial-to-mesenchymal transition (EndMT), a phenotypic shift characterised by increased cellular motility and acquisition of mesenchymal-like features. Notably, silencing of LOXL2 (siLOXL2) attenuated oxLDL-induced inflammatory responses and EndMT, further supporting the role of inflammation-driven phenotypic modulation in promoting endothelial migration. In addition, the treatment used, siLOXL2, inhibits the endothelial inflammatory response and EndMT induced by oxLDL [43].

In the present study, we postulate that the reduction in oxLDL-induced migration following SQTRF, TRF, and statin treatments may be attributed to modulation of inflammatory signalling pathways, particularly NF-κB activation and downstream adhesion molecules such as ICAM-1 and VCAM-1. Given the established role of NF-κB-mediated endothelial activation in regulating adhesion molecule expression, suppression of this pathway may cause the observed attenuation of endothelial migratory responses. Consistent with this hypothesis, our gene expression analysis demonstrated that oxLDL significantly upregulated NF-κB and ICAM-1, but not VCAM-1, expression compared with the control group. These findings confirm activation of inflammatory signalling pathways known to promote endothelial activation and migration. Treatment with SQTRF, TRF, and statin significantly attenuated the oxLDL-induced upregulation of these genes, suggesting that suppression of NF-κB-dependent inflammatory signalling may contribute to the reduction in endothelial migratory activity observed in this study. Given that ICAM-1 and VCAM-1 facilitate endothelial activation and leukocyte adhesion, their downregulation further supports that SQTRF modulates oxLDL-induced endothelial dysfunction at the transcriptional level.

Previous studies provide mechanistic support for these findings. Cárdeno et al. (2015) reported that squalene significantly reduced phosphorylated p65-NFκB and decreased mRNA expression of downstream pro-inflammatory mediators, including TNF-α and IL-1β in lipopolysaccharide (LPS)-treated murine peritoneal macrophages [44]. Similarly, Muid et al. (2022) demonstrated that palm-derived tocotrienol-rich fraction (TRF) inhibited NF-κB activation and reduced ICAM-1 and VCAM-1 expression in LPS-stimulated HUVECs, thus attenuating endothelial inflammation [45]. Collectively, these findings support that both squalene and TRF possess anti-inflammatory properties mediated through modulation of NF-κB signalling.

An important observation in the present study was that the endothelial phenotype induced by SQTRF alone differed from that observed when SQTRF was administered in the presence of oxLDL. In the absence of oxLDL, SQTRF alone increased endothelial viability and showed a trend towards enhanced tube formation, while simultaneously reducing endothelial was associated with changes in proliferation and, migration compared with oxLDL-treated cells. At the molecular level, SQTRF alone, and tube formation, together with increased VCAM-1, ICAM-1, and NF-κB expression mRNA relative to untreated control cells. In contrast, in oxLDL-induced endothelial cells, SQTRF treatment, particularly at the higher concentration, resulted in reduced tube formation and lower proliferative and migratory responses compared with oxLDL alone cells. These findings suggest that the effects of SQTRF can modulate endothelial cellular behaviour even under relatively basal conditions, with responses that are not uniformly anti-inflammatory. This observation is consistent with previous findings that tocotrienol can directly influence endothelial proliferation and tube formation [46].

In contrast, oxLDL establishes a substantially different cellular environment characterized by endothelial activation and dysfunction. OxLDL is known to induce endothelial responses through proatherogenic mechanisms involving oxidative stress, scavenger receptors such as lectin-like oxidized LDL receptor-1 (LOX-1), and inflammatory signalling pathways [47]. Consequently, the biological response to SQTRF in oxLDL-exposed cells may reflect modulation of an activated endothelial phenotype compared to its effects on basal endothelial function. In the present study, this distinction was evident at the molecular level, where SQTRF reduced the oxLDL-associated increase in NF-κB mRNA. The observed response under oxLDL exposure is also parallel with previous findings showing that tocotrienol-rich palm-derived preparations can preserve endothelial function particularly under oxidative stress conditions [48].

Taken together, these findings suggest that the effects of SQTRF are context-dependent rather than uniformly directed towards the same endothelial phenotype. Under basal conditions, SQTRF may influence physiological endothelial processes, meanwhile under oxLDL-induced stress, its effects may become more apparent in modulating the cellular responses associated with endothelial dysfunction. Importantly, the present study measured NF-κB at the gene-expression level does not necessarily reflect functional NF-κB activity, which is regulated predominantly at the protein level through processes such as IκB degradation, p65 phosphorylation and nu-clear translocation. Therefore, the observed reduction in NF-κB p65 mRNA cannot be directly interpreted as suppression of NF-κB signalling. Further studies assessing NF-κB protein activation, phosphorylation or nuclear translocation, together with oxidative stress parameters, would be required to clarify the precise molecular mechanism underlying this response.

### Limitations and Perspectives of This Study

Despite providing insights into the protective effects of SQTRF against oxLDL-induced endothelial dysfunction, several limitations have been acknowledged. First, this study was conducted exclusively in an in vitro HUVEC model, which, although widely used to investigate endothelial biology, does not fully recapitulate the complex hemodynamic, inflammatory, and multicellular interactions present in vivo during atherogenesis. Therefore, extrapolation of these findings to whole-organism physiology should be approached with caution. Future studies using more complex experimental models, including co-culture systems, genetically manipulated or transfected endothelial cells, and in vivo models, could provide further validation of the observed effects and clarify the underlying molecular mechanisms.

Second, while our data demonstrate modulation of NF-κB and adhesion molecule gene expression, the present study did not directly assess upstream or related signalling events such as ROS generation, antioxidant enzyme activity, or NF-κB nuclear translocation or protein expression. Although the antioxidant properties of tocotrienols are well established, these parameters were beyond the primary scope of the present study, which focused on the inflammatory component of oxLDL-induced endothelial dysfunction. In particular, the absence of protein-level validation limits the extent to which the observed changes in gene expression can be interpreted as evidence of altered signalling activity. Future studies should therefore evaluate oxidative stress, antioxidant defence mechanisms, and NF-κB activation at the protein level, including p65 phosphorylation status, to further strengthen mechanistic interpretation.

Third, although SQTRF demonstrated enhanced modulatory effects compared to TRF alone, the study was not specifically designed to establish pharmacological synergy. A formal evaluation of synergistic interactions would require systematic dose–response analyses and combination index assessments, which were beyond the scope of the present study. In addition, isolated palm-derived squalene was not available for independent testing, as squalene obtained from this plant source exhibits stability challenges when extracted and stored as a single compound. Consequently, direct comparison between purified squalene and the combined formulation could not be performed.

Additionally, angiogenesis was assessed using a tube formation assay, which represents an early and simplified model of capillary network formation. Incorporating complementary assays such as spheroid sprouting, 3D co-culture systems, or in vivo models (i.e., Matrigel plug assay or ApoE knockout mouse model of atherosclerosis) provide more comprehensive validation of the anti-angiogenic effects observed.

Furthermore, the molecular analyses of VCAM-1, ICAM-1, and NF-κB were performed using only the selected SQTRF concentration and therefore do not provide a complete concentration-response assessment of the molecular effects of SQTRF. Consequently, the molecular findings cannot be directly extrapolated to the higher SQTRF concentration that produced the most pronounced inhibition of tube formation. Future studies incorporating concentration-dependent molecular analyses will be important to determine whether the differential functional responses are associated with corresponding changes in endothelial inflammatory and signalling pathways.

Future investigations should also explore the impact of SQTRF on additional markers of endothelial function, including eNOS activity, nitric oxide bioavailability, oxidative stress markers, and inflammatory cytokine secretion. Given the central role of NF-κB in atherogenesis, deeper exploration of redox-sensitive signalling pathways and endothelial-to-mesenchymal transition (EndMT) markers would further clarify the molecular mechanisms underlying SQTRF-mediated vascular protection. Collectively, the present findings suggest that SQTRF modulates inflammatory-associated gene expression and endothelial functional responses in an oxLDL-induced endothelial dysfunction model. Further mechanistic and in vivo studies are warranted to substantiate its therapeutic relevance in the prevention or attenuation of atherosclerosis.

## 5. Conclusions

SQTRF attenuated oxLDL-induced endothelial dysfunction by preserving cell viability, suppressing NF-κB-mediated inflammatory gene expression, and modulating pathological angiogenic responses. These findings suggest that SQTRF exerts protective effects against early endothelial activation associated with atherogenesis.

## Figures and Tables

**Figure 1:**
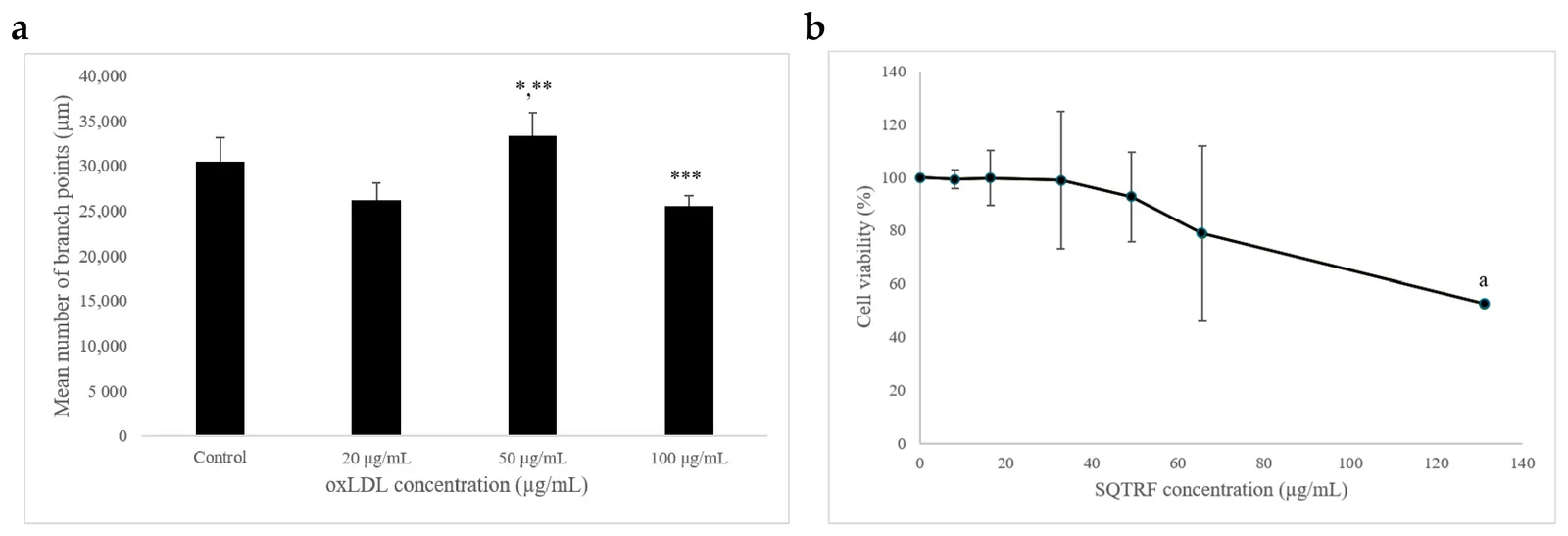
Concentration optimisation for oxLDL and SQTRF. (**a**) Effects of oxLDL (20–100 µg/mL) on endothelial tube formation in HUVECs after 24 h exposure. (**b**) Effects of SQTRF (8.2–131.2 µg/mL) on the viability of HUVECs after 24 h exposure * *p* < 0.05 compared with 100 µg/mL group, ** *p* < 0.05 compared with 20 µg/mL group *** compared with control group, ^a^ *p* < 0.05 compared with SQTRF concentrations of 8.2, 16.4, 32.8, 49.2, and 65.6 µg/mL. Data are presented as mean ± standard deviation from three independent experiments (n = 3).

**Figure 2:**
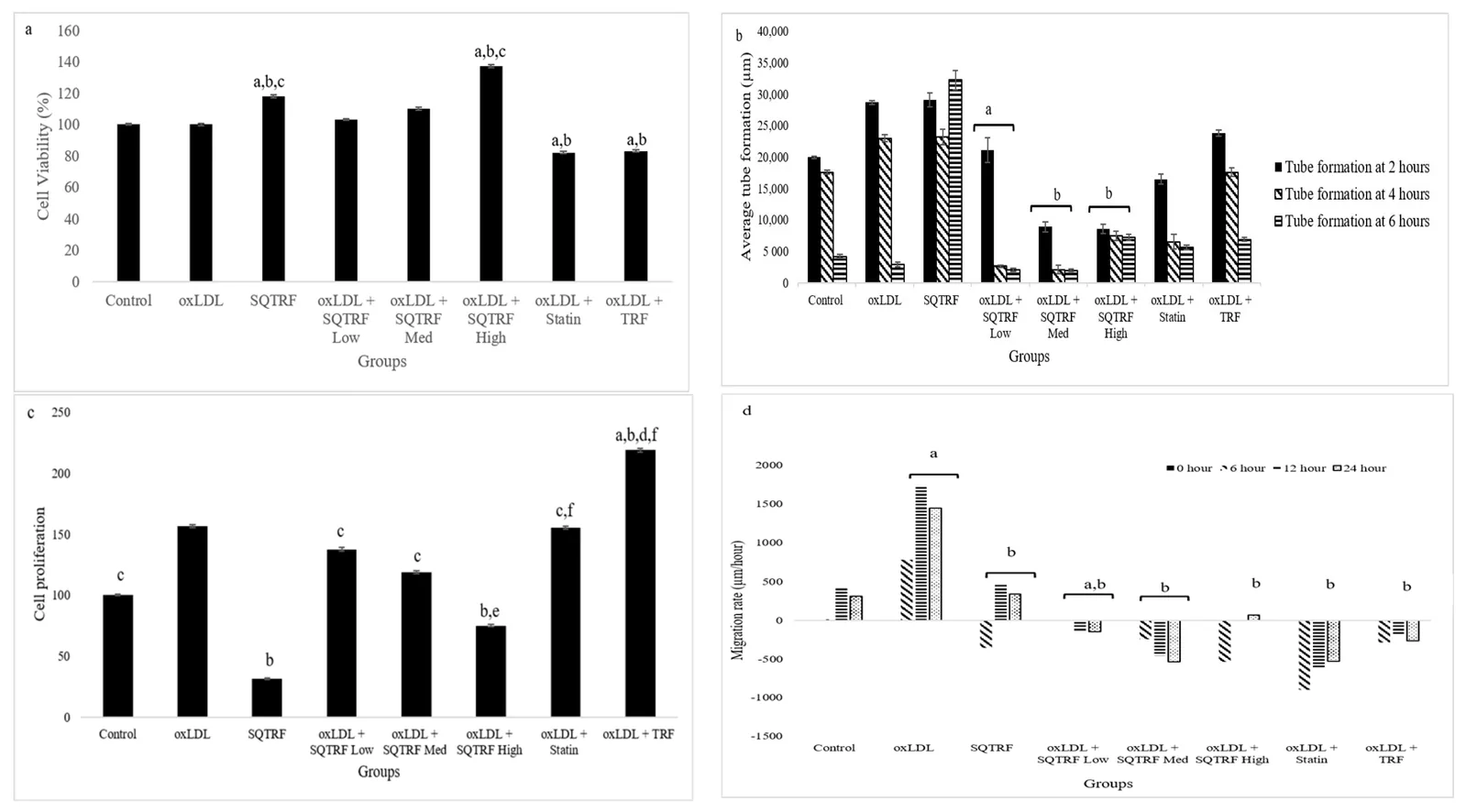
Effects of SQTRF on oxLDL-induced endothelial dysfunction in HUVECs. (**a**) Cell viability of HUVECs following treatment with 50 μg/mL oxLDL and SQTRF for 24 h. ^a^ *p* < 0.05 compared with the control group, ^b^ *p* < 0.05 compared to the oxLDL group, ^c^ *p* < 0.05 compared to the oxLDL + atorvastatin and oxLDL + TRF groups. (**b**) Average tube formation in HUVECs at 2, 4, and 6 h following atherogenic induction with oxLDL (50 μg/mL) for 24 h. ^a^ *p* < 0.05 compared the control group, ^b^ *p* < 0.05 compared to the oxLDL group. (**c**) Proliferation of HUVECs exposed to oxLDL (50 μg/mL) for 24 h. ^a^ *p* < 0.05 compared to the control group, ^b^ *p* < 0.05 compared to the oxLDL group, ^c^ *p* < 0.05 compared to the oxLDL + TRF group, ^d^ *p* < 0.05 compared to the oxLDL + atorvastatin group, ^e^ *p* < 0.05 compared to both oxLDL + TRF and oxLDL + statin, ^f^ *p* < 0.05 compared to the oxLDL + SQTRF 49.2 μg/mL group. (**d**) Migration rate measured at 6, 12, and 24 h in HUVECs induced for atherogenesis by oxLDL (50 μg/mL) for 24 h. ^a^ *p* < 0.05 compared the control group, ^b^ *p* < 0.05 compared to the oxLDL group. Control (untreated); oxLDL (oxLDL alone); SQTRF (49.2 μg/mL alone); oxLDL + SQTRF Low (16.4 μg/mL); oxLDL + SQTRF Med (32.8 μg/mL); oxLDL + SQTRF High (49.2 μg/mL); oxLDL + Statin (10 μM atorvastatin); oxLDL + TRF (4.11 μg/mL). Data are presented as mean ± standard deviation from three independent experiments (n = 3).

**Figure 3:**
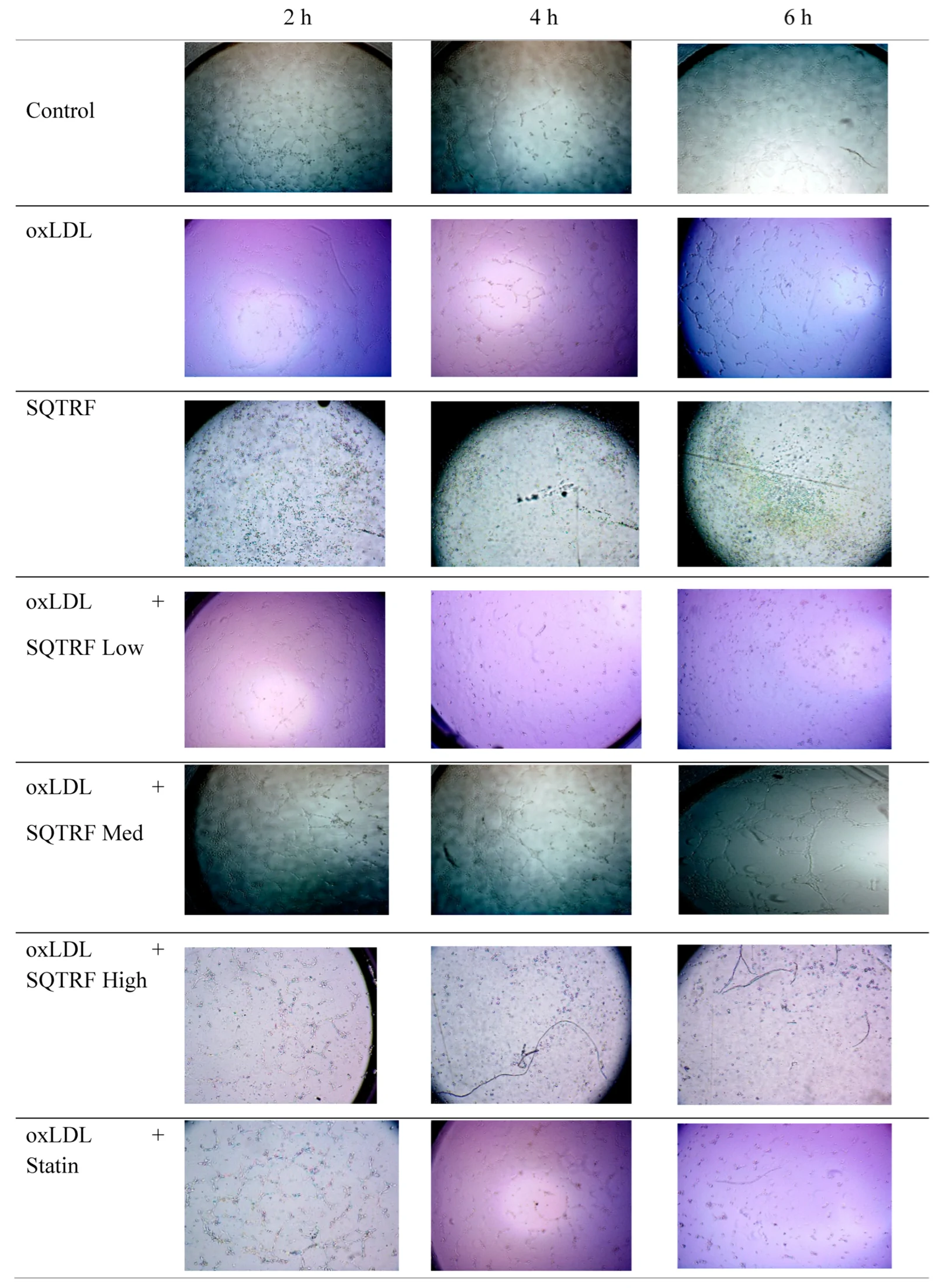

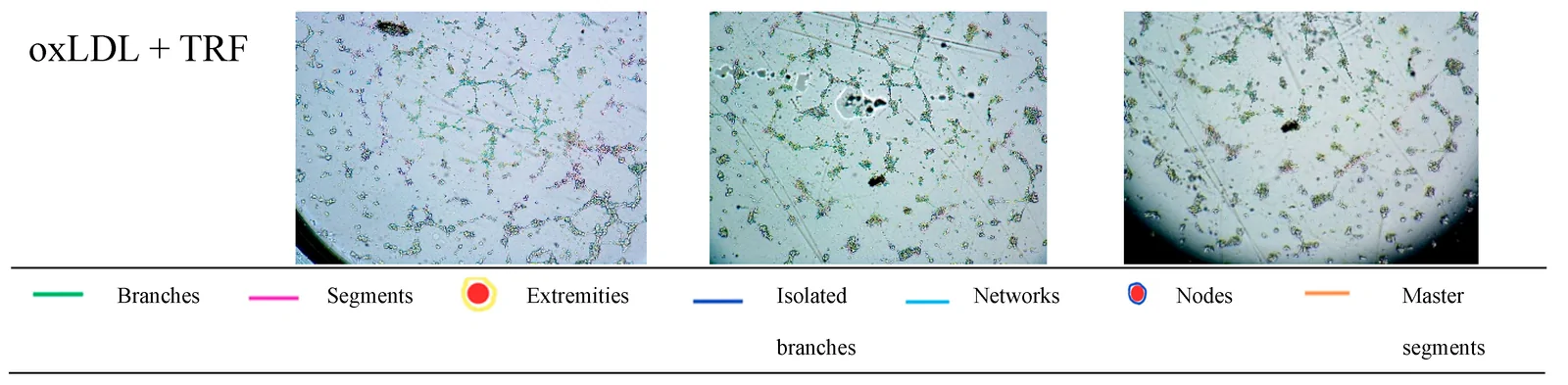
Representative images of capillary-like tube formation by HUVECs following treatment with oxLDL, SQTRF, or atorvastatin after 2 h, 4 h, and 6 h of incubation. Tube structures were visualised using an inverted phase-contrast microscope (100× magnification) and analysed using the ImageJ Angiogenesis Analyzer plugin. Colour-coded annotations indicate branches, segments, junctions, extremities, meshes, and master segments generated by the analysis software.

**Figure 4:**
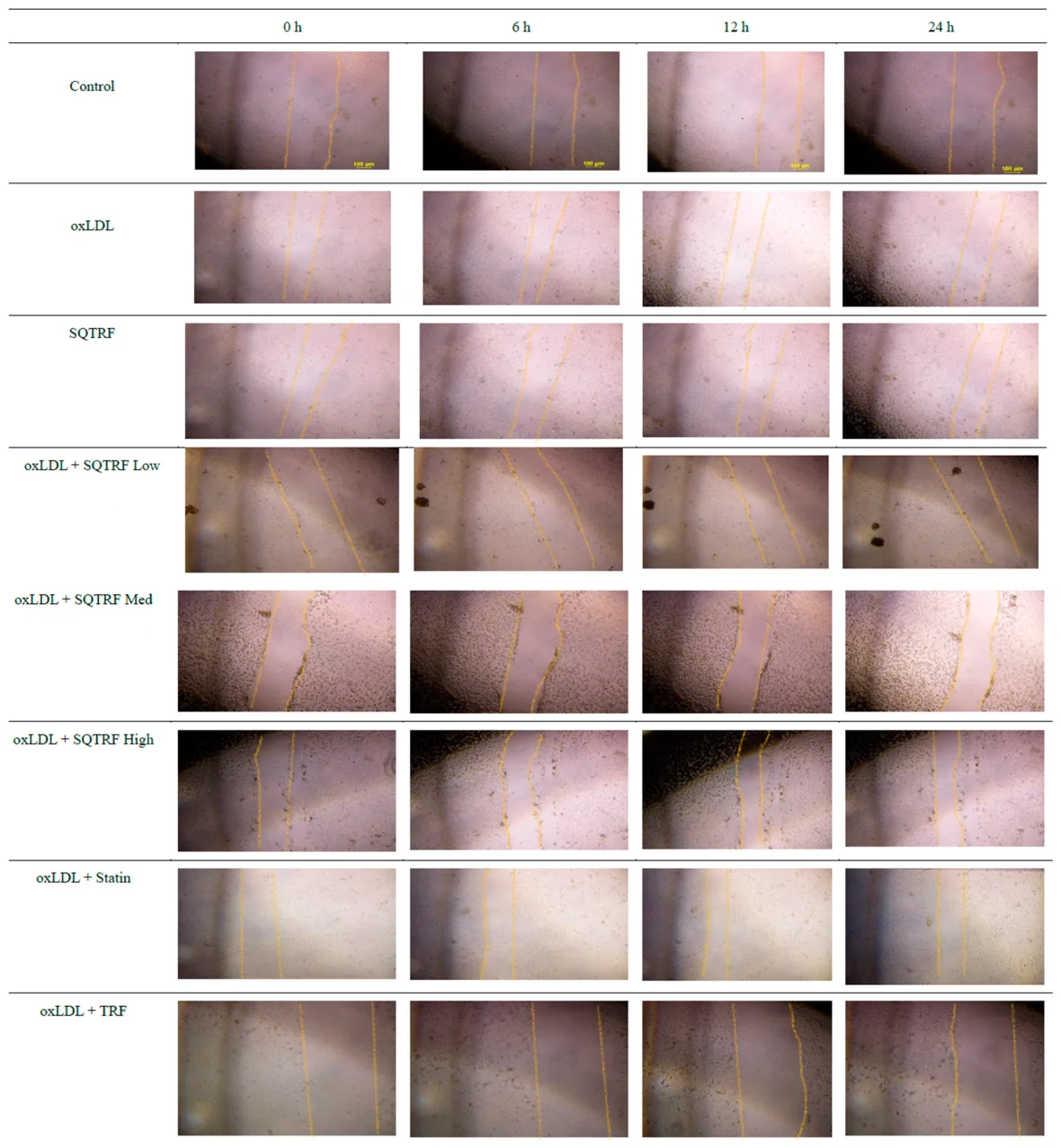
Representative images of scratch-wound migration in HUVECs following treatment with oxLDL, SQTRF, or atorvastatin 0, 6, 12, and 24 h after scratch creation. Images were captured using an inverted phase-contrast microscope at 200× magnification. The annotated lines indicate the wound edges used to define the wound boundaries for migration analysis. Wound closure was quantified using ImageJ software by measuring the change in wound area over time.

**Figure 5:**
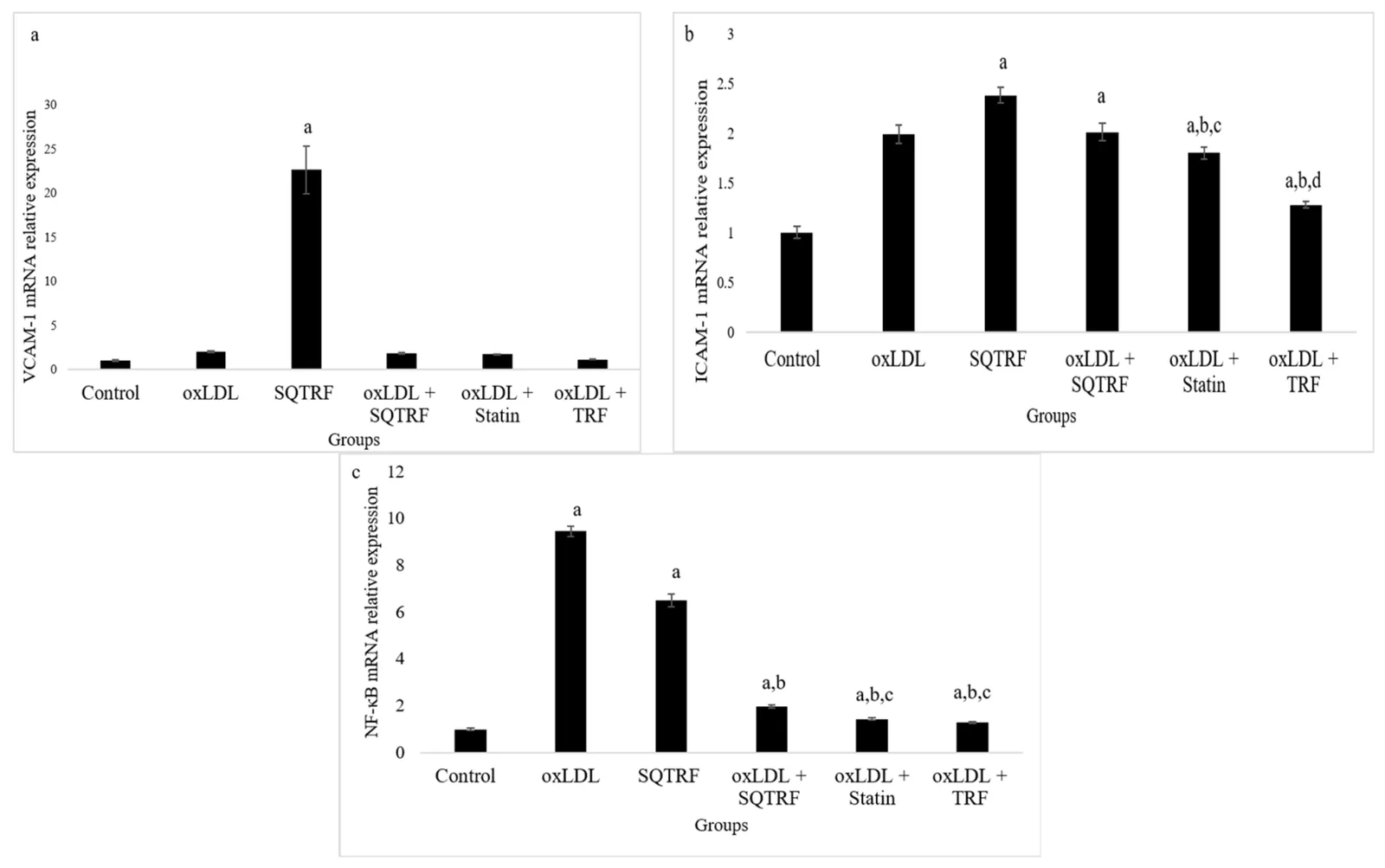
Effects of SQTRF on inflammatory gene expression in oxLDL-stimulated HUVECs. (**a**) Relative VCAM-1 mRNA expression in a HUVEC model of atherogenesis. (**b**) Relative ICAM-1 mRNA expression in a HUVEC model of atherogenesis. (**c**) Relative NF-κB mRNA expression in a HUVEC model of atherogenesis. ^a^ *p* < 0.05 compared to the control group, ^b^ *p* < 0.05 compared to the oxLDL group, ^c^ *p* < 0.05 compared to the oxLDL + SQTRF group, ^d^ *p* < 0.05 compared to oxLDL + atorvastatin group. Control (untreated); oxLDL (oxLDL alone); SQTRF (49.2 μg/mL alone); oxLDL + SQTRF (32.8 µg/mL); oxLDL + Statin (10 μM atorvastatin); oxLDL + TRF (4.11 μg/mL). Data are presented as mean ± standard deviation from three independent experiments (n = 3).

**Table 1:** Primer sequences used for qRT-PCR.

Target Gene	GenBank Accession No.	Forward Primer (5′–3′)	Reverse Primer (5′–3′)	Amplicon Size (bp)
ICAM-1	NM_000201	CAGTCACCTATGGCAACGACT	CTCTGGCTTCGTCAGAATCAC	179
VCAM-1	NM_001078	AGTTGAAGGATGCGGGAGTAT	GGATGCAAAATAGAGCACGAG	143
NF-κB p65	NM_003998	AGTGCAGAGGAAACGTCAGAA	CATTTTACCACTTGGCAGGAA	163
GAPDH	NM_002046	TCCCTGAGCTGAACGGGAAG	GGAGGAGTGGGTGTCGCTGT	217

## Data Availability

The data presented in this study are available from the corresponding author upon reasonable request, subject to institutional restrictions related to the ongoing patent application process.

## References

[1] World Health Organization Cardiovascular Diseases (CVDs). https://www.who.int/en/news-room/fact-sheets/detail/cardiovascular-diseases-(cvds).

[2] Nedkoff L., Briffa T., Zemedikun D., Herrington S., Wright F.L. (2023). Global Trends in Atherosclerotic Cardiovascular Disease. Clin. Ther..

[3] Yan R., Zhang X., Xu W., Li J., Sun Y., Cui S., Xu R., Li W., Jiao L., Wang T. (2024). ROS-Induced Endothelial Dysfunction in the Pathogenesis of Atherosclerosis. Aging Dis..

[4] Higashi Y. (2022). Roles of Oxidative Stress and Inflammation in Vascular Endothelial Dysfunction-Related Disease. Antioxidants.

[5] Thangasparan S., Kamisah Y., Ugusman A., Anuar N.N.M., Ibrahim N. (2024). Unravelling the Mechanisms of Oxidised Low-Density Lipoprotein in Cardiovascular Health: Current Evidence from In Vitro and In Vivo Studies. Int. J. Mol. Sci..

[6] Jiang H., Zhou Y., Nabavi S.M., Sahebkar A., Little P.J., Xu S., Weng J., Ge J. (2022). Mechanisms of Oxidized LDL-Mediated Endothelial Dysfunction and Its Consequences for the Development of Atherosclerosis. Front. Cardiovasc. Med..

[7] Toshima S., Hasegawa A., Kurabayashi M., Itabe H., Takano T., Sugano J., Shimamura K., Kimura J., Michishita I., Suzuki T. (2000). Circulating oxidized low density lipoprotein levels. A biochemical risk marker for coronary heart disease. Arterioscler. Thromb. Vasc. Biol..

[8] He X.M., Xiang D.C., He J.X., Hong C.J., Qiu J., Ma J., Zhang J.X. (2007). Comparison of plasma low-density lipoprotein and oxidized low-density lipoprotein levels with coronary lesion severities in patients with coronary artery disease. Zhonghua Xin Xue Guan Bing. Za Zhi.

[9] Verhoye E., Langlois M.R. (2009). Circulating oxidized low-density lipoprotein: A biomarker of atherosclerosis and cardiovascular risk?. Clin. Chem. Lab. Med..

[10] Tian K., Ogura S., Little P.J., Xu S., Sawamura T. (2019). Targeting LOX-1 in atherosclerosis and vasculopathy: Current knowledge and future perspectives. Ann. N. Y. Acad. Sci..

[11] Zhong L., Simard M.J., Huot J. (2018). Endothelial microRNAs regulating the NF-κB pathway and cell adhesion molecules during inflammation. FASEB J..

[12] Zhang M., Xu Y., Jiang L. (2019). Irisin attenuates oxidized low-density lipoprotein impaired angiogenesis through AKT/mTOR/S6K1/Nrf2 pathway. J. Cell. Physiol..

[13] Zhang C., Adamos C., Oh M.-J., Baruah J., Ayee M.A.A., Mehta D., Wary K.K., Levitan I. (2017). oxLDL induces endothelial cell proliferation via Rho/ROCK/Akt/p27(kip1) signaling: Opposite effects of oxLDL and cholesterol loading. Am. J. Physiol. Cell Physiol..

[14] Liang G., Chen S., Xin S., Dong L. (2021). Overexpression of hsa_circ_0001445 reverses oxLDL-induced inhibition of HUVEC proliferation via SRSF1. Mol. Med. Rep..

[15] Lou-Bonafonte J.M., Martínez-Beamonte R., Sanclemente T., Surra J.C., Herrera-Marcos L.V., Sanchez-Marco J., Arnal C., Osada J. (2018). Current Insights into the Biological Action of Squalene. Mol. Nutr. Food Res..

[16] Ibrahim N., Mohamed I.N. (2021). Interdependence of Anti-Inflammatory and Antioxidant Properties of Squalene-Implication for Cardiovascular Health. Life.

[17] Popa O., Băbeanu N.E., Popa I., Niță S., Dinu-Pârvu C.E. (2015). Methods for obtaining and determination of squalene from natural sources. Biomed Res. Int..

[18] Nang Lau H.L., Puah C.W., Choo Y.M., Ma A.N., Chuah C.H. (2005). Simultaneous quantification of free fatty acids, free sterols, squalene, and acylglycerol molecular species in palm oil by high-temperature gas chromatography-flame ionization detection. Lipids.

[19] Ibrahim N., Fairus S., Zulfarina M.S., Naina Mohamed I. (2020). The Efficacy of Squalene in Cardiovascular Disease Risk-A Systematic Review. Nutrients.

[20] Aggarwal B.B., Sundaram C., Prasad S., Kannappan R. (2010). Tocotrienols, the vitamin E of the 21st century: Its potential against cancer and other chronic diseases. Biochem. Pharmacol..

[21] Ibrahim N., Mohamed N., Soelaiman I.N., Shuid A.N. (2015). The Effects of Targeted Deliveries of Lovastatin and Tocotrienol on Ossification-Related Gene Expressions in Fracture Healing in an Osteoporosis Rat Model. Int. J. Environ. Res. Public Health.

[22] Rafique S., Khan D.A., Farhat K., Khan M.A., Noor M., Sharif M. (2024). Comparative efficacy of tocotrienol and tocopherol (vitamin E) on atherosclerotic cardiovascular diseases in humans. J. Pak. Med. Assoc..

[23] Trugilho L., Alvarenga L., Cardozo L., Paiva B., Brito J., Barboza I., Almeida J., dos Anjos J., Khosla P., Ribeiro-Alves M. (2025). Effects of Tocotrienol on Cardiovascular Risk Markers in Patients With Chronic Kidney Disease: A Randomized Controlled Trial. J. Nutr. Metab..

[24] Relas H., Gylling H., Miettinen T.A. (2000). Dietary squalene increases cholesterol synthesis measured with serum non-cholesterol sterols after a single oral dose in humans. Atherosclerosis.

[25] Fairus S., Nor R.M., Cheng H.M., Sundram K. (2012). Alpha-tocotrienol is the most abundant tocotrienol isomer circulated in plasma and lipoproteins after postprandial tocotrienol-rich vitamin E supplementation. Nutr. J..

[26] Zahara A.M., Lee C.C., Fatimah I.S., Poh B.K., Khairul O., Das S., Mohamad J. (2010). Plasma vitamin C and tocotrienols changes in response to dietary supplementation among young male adults. Clin. Ther..

[27] Suhaila R.N., Safuan S. (2017). Isolation Methods and Culture Conditions of Human Umbilical Vein Endothelial Cells from Malaysian Women. Sains Malays..

[28] Ugusman A., Zakaria Z., Hui C.K., Nordin N.A.M.M. (2010). Piper sarmentosum increases nitric oxide production in oxidative stress: A study on human umbilical vein endothelial cells. Clinics.

[29] Das B., Yeger H., Baruchel H., Freedman M., Koren G., Baruchel S. (2003). In vitro cytoprotective activity of squalene on a bone marrow versus neuroblastoma model of cisplatin-induced toxicity. implications in cancer chemotherapy. Eur. J. Cancer.

[30] Weng-Yew W., Selvaduray K.R., Ming C.H., Nesaretnam K. (2009). Suppression of tumor growth by palm tocotrienols via the attenuation of angiogenesis. Nutr. Cancer.

[31] Hernández-Perera O., Pérez-Sala D., Navarro-Antolín J., Sánchez-Pascuala R., Hernández G., Díaz C., Lamas S. (1998). Effects of the 3-hydroxy-3-methylglutaryl-CoA reductase inhibitors, atorvastatin and simvastatin, on the expression of endothelin-1 and endothelial nitric oxide synthase in vascular endothelial cells. J. Clin. Investig..

[32] Jiang J., Hiron T.K., Chalisey A., Malhotra Y., Agbaedeng T., O’cAllaghan C.A. (2025). Ox-LDL induces a non-inflammatory response enriched for coronary artery disease risk in human endothelial cells. Sci. Rep..

[33] Zhu Z., Li J., Tong R., Zhang X., Yu B. (2022). Astaxanthin suppresses End MT by LOX-1 pathway in ox-LDL-induced HUVECs. Eur. J. Inflamm..

[34] He W., Zhao L., Wang P., Ren M., Han Y. (2025). MiR-125b-5p ameliorates ox-LDL-induced vascular endothelial cell dysfunction by negatively regulating TNFSF4/TLR4/NF-κB signaling. BMC Biotechnol..

[35] Yu S., Wong S.L., Lau C.W., Huang Y., Yu C.-M. (2011). Oxidized LDL at low concentration promotes in-vitro angiogenesis and activates nitric oxide synthase through PI3K/Akt/eNOS pathway in human coronary artery endothelial cells. Biochem. Biophys. Res. Commun..

[36] Camaré C., Trayssac M., Garmy-Susini B., Mucher E., Sabbadini R., Salvayre R., Negre-Salvayre A. (2015). Oxidized LDL-induced angiogenesis involves sphingosine 1-phosphate: Prevention by anti-S1P antibody. Br. J. Pharmacol..

[37] Guo S., Lok J., Liu Y., Hayakawa K., Leung W., Xing C., Ji X., Lo E.H. (2014). Assays to examine endothelial cell migration, tube formation, and gene expression profiles. Methods Mol. Biol..

[38] Liu X., Zhang J., Yi T., Li H., Tang X., Liu D., Wu D., Li Y. (2025). Decoding tumor angiogenesis: Pathways, mechanisms, and future directions in anti-cancer strategies. Biomark. Res..

[39] Hou X.-Z., Yang Y.-T., Yao J.-M. (2026). Vulnerable plaques in atherosclerosis: Focus on angiogenesis-associated phenotypic crosstalk. Front. Pharmacol..

[40] Mitsos S., Katsanos K., Koletsis E., Kagadis G.C., Anastasiou N., Diamantopoulos A., Karnabatidis D., Dougenis D. (2012). Therapeutic angiogenesis for myocardial ischemia revisited: Basic biological concepts and focus on latest clinical trials. Angiogenesis.

[41] Khor H., Chieng D. (1997). Effect of squalene, tocotrienols and α-tocopherol supplementations in the diet on serum and liver lipids in the hamster. Nutr. Res..

[42] Sluiter T.J., van Buul J.D., Huveneers S., Quax P.H.A., de Vries M.R. (2021). Endothelial Barrier Function and Leukocyte Transmigration in Atherosclerosis. Biomedicines.

[43] Ma J., Ling J., Tong R., Guo J., Zhu Z. (2024). siLOXL2 Inhibits Endothelial Inflammatory Response and EndMT Induced by ox-LDL. Cerebrovasc. Dis. Extra.

[44] Cárdeno A., Aparicio-Soto M., Montserrat-de la Paz S., Bermudez B., Muriana F.J., Alarcón-De-La-Lastra C. (2015). Squalene targets pro- and anti-inflammatory mediators and pathways to modulate over-activation of neutrophils, monocytes and macrophages. J. Funct. Foods.

[45] Abd Muid S., Froemming G.A.R., Ali A.M., Rahman T.H.A., Hamid Z., Nawawi H. (2022). Effects of palm oil derived tocotrienol rich fraction and vitamin E isomers on biomarkers of early atherogenesis in stimulated human umbilical vein endothelial cells. Malays. Appl. Biol..

[46] Inokuchi H., Hirokane H., Tsuzuki T., Nakagawa K., Igarashi M., Miyazawa T. (2003). Anti-angiogenic Activity of Tocotrienol. Biosci. Biotechnol. Biochem..

[47] Pirillo A., Norata G.D., Catapano A.L. (2013). LOX-1, OxLDL, and atherosclerosis. Mediat. Inflamm..

[48] Ali S.F., Woodman O.L. (2015). Tocotrienol Rich Palm Oil Extract Is More Effective Than Pure Tocotrienols at Improving Endothelium-Dependent Relaxation in the Presence of Oxidative Stress. Oxidative Med. Cell. Longev..

